# Inclusion of palliative and end of life care in health strategies aimed at integrated care: a documentary analysis [version 2; peer review: 2 approved]

**DOI:** 10.12688/amrcopenres.13079.2

**Published:** 2023-01-10

**Authors:** Rachel L. Chambers, Sophie Pask, Irene J. Higginson, Stephen Barclay, Fliss E.M. Murtagh, Katherine E. Sleeman

**Affiliations:** 1 Cicely Saunders Institute of Palliative Care, Policy & Rehabilitation, King’s College, London, SE5 9PJ, UK; 2 Wolfson Palliative Care Research Centre, Hull York Medical School, University of Hull, Hull, UK; 3 King’s College Hospital NHS Foundation Trust, Denmark Hill, London, UK; 4 Department of Public Health and Primary Care, University of Cambridge, Cambridge, UK

**Keywords:** Palliative, End of Life Care, Integrated Care Systems, Health Policy, Documentary Analysis

## Abstract

**Background:**

In England, Integrated Care Systems have been established to improve integration of care, as part of the NHS Long Term Plan. For people near the end of life, palliative care can improve integration of care. We aimed to understand whether and how palliative and end of life care was included in Integrated Care System strategies, and to consider priorities for strengthening this.

**Methods:**

Documentary analysis of Integrated Care System (ICS) strategies, using summative content analysis, was performed. Google searches were used to identify NHS Trust, Clinical Commissioning Group or ICS websites. We searched these websites to identify strategies. Key terms were used to identify relevant content. Themes were mapped onto an adapted logic model for integrated care.

**Results:**

23 Integrated Care System strategy documents were identified. Of these, two did not mention any of the key terms, and six highlighted palliative and end of life care as either a priority, area of focus, or an ambition. While most (19/23) strategies included elements that could be mapped onto the adapted logic model for integrated care, the thread from enablers and components, to structures, processes, outcomes, and impact was incomplete.

**Conclusions:**

Greater prioritisation of palliative and end of life care within recently established Integrated Care Systems could improve outcomes for people near the end of life, as well as reduce reliance on acute hospital care. Integrated Care Systems should consider involving patients, the public and palliative care stakeholders in the ongoing development of strategies. For strategies to be effective, our adapted logic model can be used to outline how different components of care fit together to achieve defined outcomes and impact.

## Introduction

The World Health Organisation defines integrated healthcare as ‘the organisation and management of health services so that people get the care they need, when they need it, in ways that are user-friendly, achieve the desired result and provide value for money’^1^. Integrated healthcare aims to ensure patients’ needs are addressed across services and settings^2^. Integrated care is a common policy goal and has been shown to improve ‘quality of life, service coordination, efficiency and satisfaction with care’^2,3^.

Palliative and end of life care is an important part of the solution to delivering integrated care to people living with life-limiting illnesses and their families^4^. Palliative care improves outcomes for patients, including depression and quality of life^5^. Notably, palliative care is one of the few interventions found to reduce reliance on acute hospital care^6–8^, and increases the likelihood of patients dying at home, if that is their preference^9^.

In England, the NHS Long Term Plan (2019) aimed to improve the integration of care through the establishment of Integrated Care Systems (ICSs)^10^. ICSs were non-statutory partnerships between NHS commissioners, local authorities and other stakeholders who planned health and provided services within specified geographical areas^11^. They aimed to improve the health of their populations by offering coordinated and efficient services^11^. In total, 42 ICSs were established, which cover the whole geographical area of England (each with a population size of approximately 1–3 million people)^11^. ICSs became statutory organisations in July 2022. While there are good reasons for ICSs to include palliative and end of life care in their strategies, previous research has found that palliative care is infrequently a policy priority^4, 12^.

We aimed to understand whether and how palliative and end of life care was included in ICS strategy documents, and to consider priorities for strengthening this. Searches for ICS strategy documents were completed on 30^th^ November 2021.

## Methods

### Study design

This study was a documentary analysis using summative content analysis to examine whether and how palliative and end of life care is included in ICS strategy documents, and provide priorities for strengthening this. In summative content analysis, systematic searches are used to quantify the occurrence of specific words or phrases^13^. This then forms the basis for more in-depth exploration of the data, for example examination of the contextual use of these words and phrases^13^. Summative content analysis is particularly useful when the aim is to summarise the content of documentary data, including policies^12^.

### Data acquisition

We identified all available ICS strategy documents. Google searches were used to identify ICS websites. Google key terms were the name of each area followed by ‘Integrated Care System’. For example, ‘Bedfordshire, Luton and Milton Keynes Integrated Care System’. We included Clinical Commissioning Group (CCG) and NHS Trust websites where ICS websites were not yet established. The web addresses of ICS, CCG and NHS websites are in Appendix 1.

On the websites we located strategy documents using the search bar and the following key terms ‘plan’ or ‘strategy’ or ‘strategies’. We also used the menu to search for a ‘publications’ page and screened the results for strategies. Searches were completed on 30^th^ November 2021.

We included strategies if they explicitly mentioned the ICS or if the documents referred to an ICS. We included five-year plans, long term plans and service strategies. We excluded ‘Sustainability and Transformation Plans’ as these were introduced in 2016 (and were replaced by the development of Integrated Care Systems).

### Data extraction and analysis

Once the strategy documents had been identified, we electronically searched each document using Acrobat Reader DC (version 2022.001.20169) and screened for the use of key terms to identify content related to palliative and end of life care. The key terms were palliat*, end of* (to capture *‘end of life’* and *‘end of their lives’*), terminal, hospice, bereave*, death, die, and dying, EoL and ‘of life’ (to capture *‘last days/months/ years of* life’). The key terms were based on those used in previous studies^4,12^. Where key terms were identified, they were highlighted in the text, and the relevant section of the strategy document was printed to form a resource folder from which familiarisation of data occurred.

We used a data extraction spreadsheet (underlying data) to record information on the name of the ICS, start and end year of the strategy document, total number of pages in the document, number of times each key term was mentioned in a relevant context (we excluded mentions of key words in non-relevant contexts, e.g. ‘accidental deaths’, ‘premature deaths/suicides’, ‘winter deaths’, or mentions of the terms only in the context of the life course e.g. ‘from birth to end of life’), whether the ICS prioritised palliative and end of life care (this included any chapters or sections devoted to this topic or any explicit mention of priority status), and whether the key terms were mentioned in specific clinical populations (e.g. cardiovascular disease, dementia etc.). Data were extracted by N.J, J.J and R.L.C.

To guide the content analysis, we used and adapted the Social Care Institute for Excellence (SCIE) logic model for integrated care^14^. We selected this model because it was designed to provide a structure for integrated health and care systems to support planning, performance monitoring, and system improvement^14^. The original logic model comprised 1) enablers, 2) components, 3) outcomes and 4) impacts. Enablers are described as ‘contextual factors that create the pre-conditions for integrated care’^14^. Components of integrated care are described as ‘interventions or activities that create integration’^14^. Outcomes reflect the effect of healthcare to improve the health status and wellbeing of patients and informal carers^15^. Impact is defined by the Social Care Institute for Excellence as ‘long-term benefits that [reflect] the convention of the Triple Aim^16^ − improving health and wellbeing, enhancing quality and providing best value care’^17^. We adapted this model to include Donabedian’s structures and processes^18^ to extract further detail on the physical components necessary to deliver care and how care is provided and received^15^. Processes are defined by Donabedian^15^ as descriptions of how care is provided and received and the ways in which structures are used. Structures are defined by Donabedian as the physical components necessary to deliver care^15^.

For the analysis, a virtual whiteboard was created using Microsoft Whiteboard (version 22.10622.110). Any mention of the key terms was extracted onto a virtual sticky note. An iterative process of discussion and analysis of each strategy informed the mapping of relevant content onto the adapted logic model. Themes and subthemes were then refined in line with the study aims. Initial coding was undertaken by R.L.C. and K.E.S. Discrepancies were resolved in discussion with F.E.M.M and S.P.

## Results

**Figure 1:**
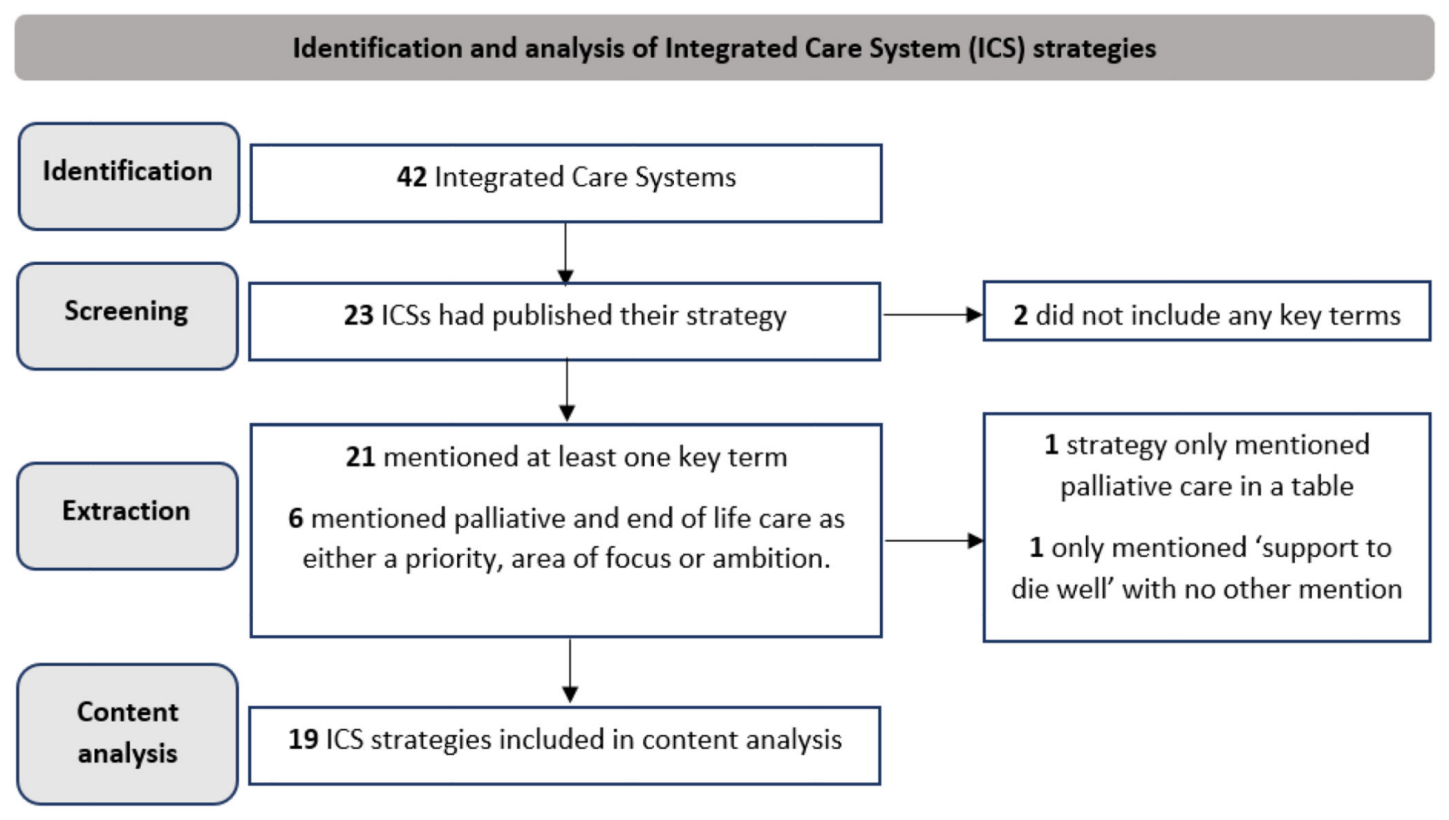
Identification and analysis of Integrated Care System strategies.

**Figure 2:**
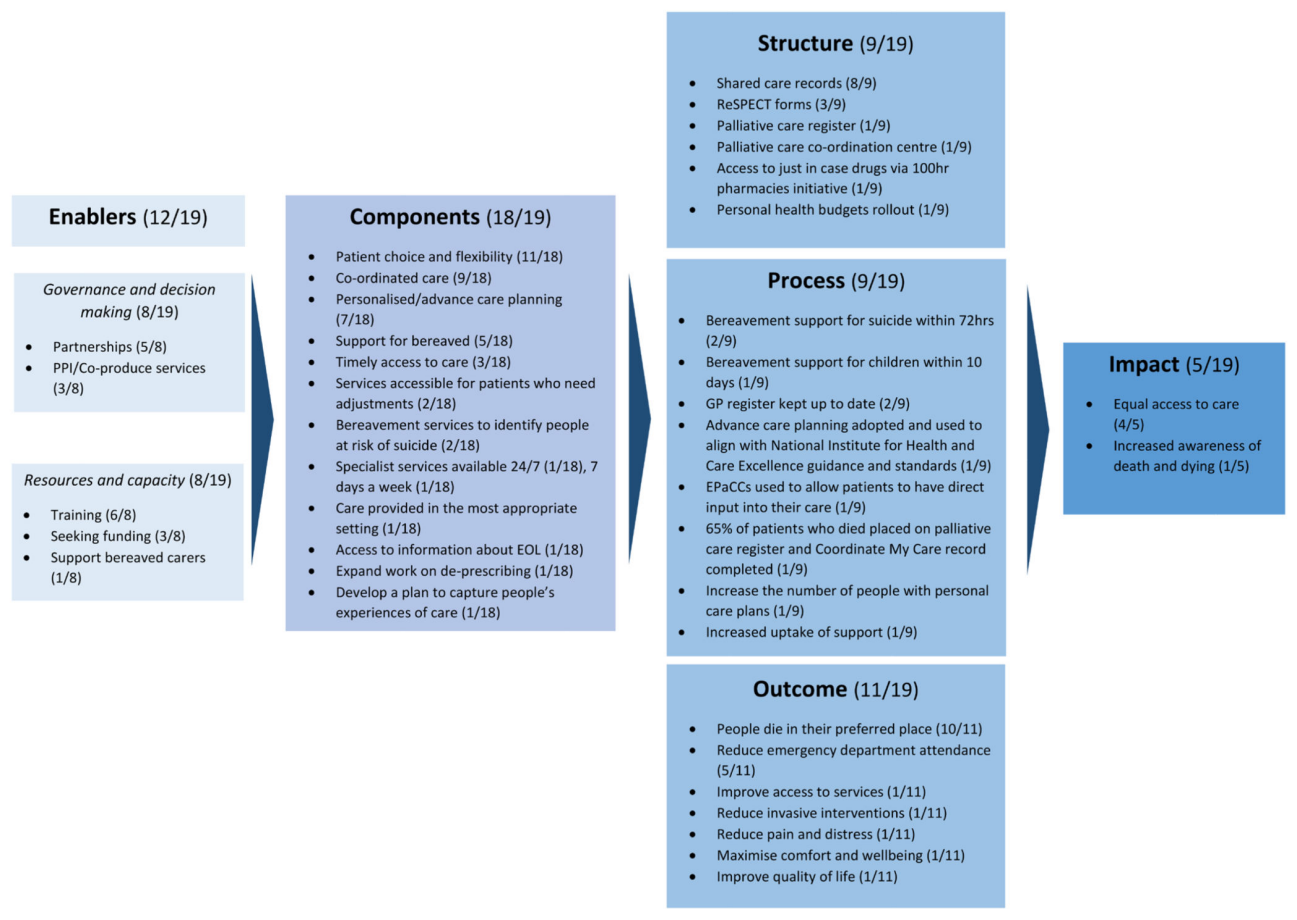
Adapted integrated care logic model.

We identified strategy documents for 23 of the 42 ICSs (on 30^th^ November 2021) published between 2015 and 2021. The 23 documents comprised a total of 2,377 pages (mean 103.3, range 12-265). Of the 23 strategy documents identified, two did not mention any of the key terms relating to palliative and end of life care (Figure 1). Therefore, 21 documents were included in the data extraction phase. The terms ‘end of their lives’ or ‘end of life’ were used most frequently (in 18 of the 21 strategies). ‘Terminal’ was used least frequently, being mentioned in only one strategy document. The term ‘bereave*’ was mentioned in 15 of the 21 strategies; in eight of these the focus was limited to bereavement after death by suicide. Six strategies specifically highlighted palliative and end of life care as either a priority, area of focus, or an ambition. Two strategies mentioned key terms but without further context. For example, one strategy included the phrase ‘palliative care’ in a table; another used the phrase ‘support to die well’, but in both cases there was no other contextual information. Therefore, 19 strategies were included in the content analysis (see Figure 2).

Of the 19 documents included in the content analysis, the number of pages per document that included at least one of the key terms ranged from 1 to 41. In most strategies, key terms were not mentioned in the context of specific clinical populations, instead palliative and end of life care was mentioned more broadly for all populations who might require it. Where key terms were associated with clinical populations, these included people with cancer (n=7), dementia (n=4), children and young people (n=3), people with respiratory disease (n=2), cardiovascular disease (n=1), diabetes (n=1), and stroke (n=1).

### Enablers

The first part of the adapted logic model comprises enablers of integrated care (see Figure 2). Enablers are described as ‘contextual factors that create the pre-conditions for integrated care’^14^. Twelve of 19 strategies included information on enablers for palliative and end of life care.

#### 1) Governance and decision-making

Eight of 19 strategies included enablers concerning governance and decision-making. Five of these eight strategies emphasised the need to work in partnership, including with hospices (5/5), voluntary end of life organisations (such as Child Bereavement UK) (3/5), primary care networks (2/5), local authorities (1/5), and academic groups (1/5) to deliver palliative and end of life care. A small number of strategies (3/8) highlighted the need to co-produce palliative and end of life services and to consult patients, families, and the public to understand their preferences for care and to discuss areas for improvement.

#### 2) Resources and capacity

Eight of 19 strategies included enablers concerning resources and capacity. Six of these eight highlighted the need to provide training and development to ensure staff have the competencies and skills to meet the needs of patients and their families/friends at the end of life. Needs identified were training in ‘sensitive conversations’, ‘person-centred care’ and how to identify when people are approaching end of life.

Three strategies specifically mentioned the need to actively seek or use funding for end of life care. One service stated that these funds would be used to ‘improve adults, children and young people hospices and palliative care services and to ensure the delivery of hospice outreach support to the community’, while another ICS had already secured funding to develop their approach to advance care planning. One strategy recognised the need to financially support carers living with loss or bereavement.

### Components

Components of integrated care are described as ‘interventions or activities that create integration’^14^. 18 of 19 ICS strategies included components relating to palliative and end of life care. Most frequently these included allowing patients to have choice and flexibility over their care (11/18) and supporting patients to make decisions based on their personal preferences through advance care planning (7/18). Five strategies included components focused on supporting people who were bereaved.

Nine of 18 strategies that included components relating to palliative and end of life care mentioned the need for co-ordinated end of life care, with liaison across services and settings including voluntary, primary, secondary, and tertiary sectors.

### Structures

Nine of 19 strategies included structures of integrated palliative and end of life care^15^. These are defined by Donabedian as the physical components necessary to deliver care^15^. Within eight strategies this included shared care records including Electronic Palliative Care Coordination Systems (EPaCCS) as urgent care planning tools. ReSPECT (Recommended summary Plan for Emergency Care and Treatment) forms^19^ were highlighted by three strategies to promote advance care planning and improve the quality of care and support patients receive. One strategy document included the use of palliative care registers as a standard identification tool for people nearing the end of life. One strategy document specifically mentioned a Palliative Care Coordination Centre to support onward referral of patients to services such as hospice at home.

### Processes

Nine of 19 strategies included processes of care. Processes are defined by Donabedian^15^ as descriptions of how care is provided and received and the ways in which structures are used. Three ICSs provided standards for bereavement care; two proposed that bereavement support for suicide should be delivered within 72 hours, while another suggested bereaved children should have access to support and information within ten days.

Three of nine strategies that included processes of care focused on care planning. One strategy document highlighted the need for all partners to adopt advance care planning to align with National Institute for Health and Care Excellence (NICE) end of life care guidance and bereavement standards. One specifically provided a target, that ‘65% patients who died should be identified by GPs and placed on GP palliative care registers and Co-ordinate My Care records completed’.

### Outcomes

Outcomes reflect the effect of healthcare to improve the health status and wellbeing of patients and informal carers^15^. Eleven of 19 strategy documents included outcomes relating to palliative and end of life care. Most highlighted the importance of allowing people to die in their preferred place (10/11). Five focused on minimising emergency department attendances. A small number of strategies mentioned additional outcomes, such as reducing pain and distress at the end of life (1/11), reducing invasive interventions (1/11), maximising comfort and wellbeing (1/11) and improving quality of life (1/11).

### Impact

Impact is defined by the Social Care Institute for Excellence as ‘long-term benefits that [reflect] the convention of the Triple Aim^16^ − improving health and wellbeing, enhancing quality and providing best value care’^17^. A minority of ICSs (5/19) described the longer-term benefits of their strategies on the population. Four of these strategies cited equal access to palliative and end of life care as a longer-term impact. For example, one strategy document mentioned that ‘regardless of borough, each patient will have access to the same level of care’. A fifth strategy cited increased community awareness of death and dying as a longer-term impact.

### Narrative thread

While most strategies included content relating to palliative and end of life care that could be mapped onto the adapted logic model, for most there was poor connection between the different elements. For example, few strategies mentioned how their enablers or components would lead to better outcomes for their patients, or the associated impact. Only two strategies included content relating to all elements of the logic model, and just one of these included a clear narrative thread from enablers to impact.

## Discussion

In this documentary analysis of published ICS strategies in England, we found that most (21/23 identified) included at least one mention of palliative and end of life care, and in six strategies palliative and end of life care was framed as a priority. While most (19/23) strategies included elements of care which could be mapped onto our adapted integrated care logic model, the thread between different elements of the logic model within individual strategies was poor.

Components of care were the most commonly included elements of the adapted logic model, in 18 of 19 strategies. Components focused on allowing patients to have choice and flexibility over their care, through advance care planning, personalised care, and care coordination. This is consistent with previous research which identified effective models of palliative care include: (i) coordination of palliative care across health and social care services; and (ii) involve patients and their families in decision making and goal setting^20^. Other components that have been identified as priorities for patients and their families, such as timely access to care and 24/7 services, were not mentioned^21^. This may reflect the lack of inclusion of patients and the public in the development of ICS strategies.

The structures that were most frequently mentioned in strategy documents were shared care records, including Electronic Palliative Care Coordination Systems (EPaCCS). EPaCCS are used across England as a way of recording and sharing information on patient preferences to enhance care coordination. EPaCCS have been shown to be useful for staff and beneficial for patients, but strong evidence for the effectiveness of these systems is currently lacking^22^.

Although most strategies included content that could be mapped onto the adapted logic model, for most there was poor connection between the different elements. For example, few strategies mentioned how their enablers or components would lead to better outcomes for their patients, or how the outcomes would be demonstrated. For strategies to be effective, clarity about how the different components fit together to achieve the desired outcomes and impact is essential^17^.

A key outcome metric for many ICSs was individuals being able to die in their preferred place. This is in keeping with previous analysis of health strategies in England^12^ and reflects a policy focus on the place of death since the 2008 End of Life Care Strategy^23^. In contrast, only a small number of ICSs mentioned outcomes such as improving pain, symptoms, or quality of life, and no strategies mentioned how these outcomes would be demonstrated. Routine use of patient-centred outcome measures can help ensure the needs of patients are met by services. By generating this data, services can show improvements in service provision and help guide future healthcare strategies and care provision^24^.

Shifting the balance of care into the community is an overarching priority for ICSs. Several strategy documents identified reducing emergency department attendances among people approaching the end of life as a key outcome. This was not apparent in previous analysis of health strategies in England, and likely reflects a more recent policy and research focus on this indicator^25^. Previous research found that 66% of all health and social care costs at the end of life are attributed to hospital activity, and emergency admissions account for 71% of hospital costs in the last year of life^26^. Integrated models of care that help to ensure patients’ needs are met and care is delivered in the most appropriate setting^26^, may also reduce the need for emergency care. This is a particularly important consideration since Emergency Department visits for people approaching the end of life are increasing^27^.

We found only a few strategies mentioned the need to ensure patients have equal access to care and outcomes at the end of life, or any actions or recommendations to address existing known inequities. Previous research has shown minority ethnic groups and individuals living in the most deprived areas receive poorer quality of end of life care^28,29^. Research has also highlighted disparities in access to palliative and end of life care services for individuals living in rural areas^30^, individuals living with learning disabilities or dementia^29,31^ and the LGBT community^32^. The importance of addressing these inequalities has been recognised in a recently published national framework for Palliative and End of Life Care 2021–2026 by the NHS in England^24^. ICSs need to recognise, acknowledge, and address inequalities and inequities to ensure all patients and their families and carers receive high quality care at the end of life, and importantly that any new initiatives do not widen the gaps. This is particularly important in light of the known projected increase in the number of people requiring palliative and end of life care in England and Wales^33^.

Several strategies mentioned the need to partner with local communities to inform them of and co-produce local palliative and end of life care services. However, there were no mention of ‘compassionate communities’ which recognise that ‘care is not a task solely for health and social care services but is everyone’s responsibility’^34^. As outlined in the Lancet commission on the Value of Death, health and social care services should work in partnership with their wider communities to provide care for people at the end of their lives^35^. *PA model of compassionate communities could be utilised by ICSs*.

Just three strategies highlighted the need to co-produce services with patients and their families/friends to enable integrated care. The importance of this has been recognised by the National Guidance for End of Life Care^36^. Individuals accessing care and receiving services should be given an opportunity to reflect and advise on how care is delivered, highlight training needs and explore how resources are used^37^. Benefits of patient and public involvement include improved service design and provision and improved clinical outcomes including quality of life^37^.

Few strategies mentioned the need for adequate funding for staff and services in palliative and end of life care. Palliative care has been shown to be a high value intervention found to reduce reliance on acute hospital care^6–8^. The literature documents that funding for palliative and end of life care needs to be a priority otherwise policy goals and recommendations may not be met^38,39^.

### Strengths and limitations

We did not include analysis of stand-alone palliative and end of life care specific strategies that might exist within the ICSs. Our analysis was completed at a time when ICSs were under formation and were developing their strategies, and it is expected that additional strategies will be published over the next few months. It is also likely that now ICSs are required to commission palliative care services, that their updated or new strategies will include references to palliative and end of life care, which may not have been captured in our analyses. The timing of research such as this is challenging; too soon risks fewer strategies to analyse, whereas too late limits the potential impact to promote changes.

### Implications

This study was designed to help guide the ongoing development of ICS strategies to strengthen provision of palliative and end of life care as part of integrated care. Our study is timely given the Health and Care Act (2022) now requires ICSs to commission palliative care services (including specialist palliative care)^40^. Our adapted logic model can be used to guide the structure of ICS strategies to strengthen commissioning of care for people approaching the end of life. Tools to guide the assessment of palliative care integration into health systems are also available^41,42^ and should be considered by local policy makers.

## Conclusions

Strengthening provision of palliative and end of life care for everyone who needs it is an evidence-based way to both improve care for people approaching the end of life and their families and friends, and to shift the balance of care into the community and reduce pressure on acute services. To support this, ICSs should prioritise and adequately resource palliative and end of life care services. A previous documentary analysis of Health and Wellbeing Strategies in England found that only half mentioned end of life care. The current paper shows some progress, with most ICS strategy documents including aspects of palliative and end of life care that could be mapped onto our adapted logic model. But for most there was incomplete and poor connection between the different elements. Involving patients and the public in the ongoing development of strategies, using our adapted logic model to ensure focus on the impacts and outcomes that are most important, and identifying evidence-informed interventions that achieve these, will help ensure high quality integrated care for people approaching the end of life. ICSs should continue to monitor palliative care integration into health systems using established tools.

## Supplementary Material

Appendix

## Data Availability

Inclusion of palliative and end of life care in health strategies aimed at integrated care: a documentary analysis.xls. http://www.doi.org/10.6084/m9.figshare.20401602^43^. Data are available under the terms of the Creative CommonsZero “No rights reserved” data waiver(CC0 1.0 Public domain dedication).

## *Revsied* Amendments from Version 1

In response to the reviewer’s comments, we have added additional detail to the discussion, reflecting on whether ICSs mention compassionate communities and the need for adequate funding for palliative and end of life care in their strategies.

In the strengths and limitations section of our paper we have added how the evolving state of ICSs affects our results.

**Any further responses from the reviewers can be found at the end of the article**

## Open Peer Review

**Current Peer Review Status:** ✔ ✔

**Version 2**

Reviewer Report 10 January 2023

https://doi.org/10.21956/amrcopenres.14240.r27041

**© 2023 Morales J et al**.This is an open access peer review report distributed under the terms of the Creative Commons Attribution License, which permits unrestricted use, distribution, and reproduction in any medium, provided the original work is properly cited.

✔ **Juan Esteban Correa Morales**http://orcid.org/0000-0001-7071-0516

^1^School of Medicine, Universidad de La Sabana, Chía, Colombia

^2^Instituto Nacional de Cancerología, Bogotá, Colombia

**Miguel Antonio Sánchez-Cárdenas**

Faculty of Nursing, Universidad El Bosque, Bogota, Colombia

We believe the authors have made the necessary changes to the text based on our feedback. In addition, we consider the paper to be outstanding and pass peer review. We don’t have any further comments to make. We would like to thank again for the opportunity of revising the document.

***Reviewer Expertise:*** Palliative care public health strategies

**We confirm that we have read this submission and believe that we have an appropriate level of expertise to confirm that it is of an acceptable scientific standard**.

**Version 1**

Reviewer Report 23 November 2022

https://doi.org/10.21956/amrcopenres.14158.r27028

**© 2022 Morales J et al**.This is an open access peer review report distributed under the terms of the Creative Commons Attribution License, which permits unrestricted use, distribution, and reproduction in any medium, provided the original work is properly cited.

?

**Juan Esteban Correa Morales** http://orcid.org/0000-0001-7071-0516

^1^ School of Medicine, Universidad de La Sabana, Chía, Colombia

^2^ Instituto Nacional de Cancerología, Bogotá, Colombia

**Miguel Antonio Sánchez-Cárdenas**

Faculty of Nursing, Universidad El Bosque, Bogota, Colombia

First of all, we want to thank the opportunity of reviewing this outstanding documentary review. No doubt palliative care integration assessment is a landmark field of research. We encounter nine suggestions for the authors’ consideration. We will not repeat points already made by the first reviewer.

1. **Methodology section:** Describe how many authors performed the data extraction.
2. **Results section:** second paragraph, second sentence: You state key terms were not usually mentioned in relation to a clinical population. We think it would be pertinent to complement this statement by giving a general idea of in what context key terms were generally mentioned in the documents.
3. **Results section:** You don’t mention if the strategies make any mention in regards to compassionate communities. It is notable to us as foreign experts that these documents don’t mention them under the governance item, given that the UK is a public health world leader and this is a milestone integration initiative. Maybe it will be worth making a comment in the discussion about this finding.
4. **Result section:** You don’t mention if any of the strategies propose a time frame to be accomplished or a follow-up process to ascertain its implementation. We think these characteristics are key to examining the strategies’ structure and an improvement opportunity you could point out in the discussion.
5. **Result section:** The definition of the impact component should be given in the methodological section.
6. **Result section:** Add the publication years of the ICSs. The time frame of publication of the documents validates their comparability and depicts possible tendencies. Knowing the document’s year of publication could provide an overview of the evolution of these documents towards or against palliative care integration. This information will also better explain your statements on the limitations section where you make a reference to the ICSs recent development albeit the document’s temporality isn’t reported. Further the information in this matter could be easily presented in a figure.
7. **Discussion section:** On the results you mention only three strategies (15%) state a funding component. The lack of a financial component is a weakness of the strategies, given it has been documented how the competition for resources and their shortage are an obstacle for the implementation of palliative care strategies. We recommend you develop/underline the implication of your result in the discussion section.(DOI: https://doi.org/10.1016/j.healthpol.2013.07.016^1^ & DOI: https://doi.org/10.1016/j.healthpol.2022.01.011^2^)
8. **Discussion section or Strength and limitations section:** We think readers will want to replicate your methodology to assess their own country’s policy level of integration. To better explain its usefulness, we think it would be ideal if you compare the pros and cons of your methodology to the ATLANTES’ proposal on palliative care integration assessment. (DOI:https://doi.org/10.1186/s12904-021-00728-z^3^ & DOI:https://doi.org/10.3390/ijerph182010753^4^)
9. **Conclusion section:** We suggest you incorporate the utmost important changes of our feedback in your conclusions.

We acknowledge the word count may limit our suggestions’ full incorporation. Please discuss and consider them as you believe they better serve your paper’s objective.

**Is the work clearly and accurately presented and does it cite the current literature?**

Yes

**Is the study design appropriate and is the work technically sound?**

Partly

**Are sufficient details of methods and analysis provided to allow replication by others?**

Yes

**If applicable, is the statistical analysis and its interpretation appropriate?**

Yes

**Are all the source data underlying the results available to ensure full reproducibility?**

Yes

**Are the conclusions drawn adequately supported by the results?**

Partly

***Reviewer Expertise:*** Palliative care public health strategies

**We confirm that we have read this submission and believe that we have an appropriate level of expertise to confirm that it is of an acceptable scientific standard, however we have significant reservations, as outlined above**.

Author Response 21 Dec 2022

**Rachel Chambers**, King’s College, London, UK

Thank you to the reviewers for their comments and feedback on our paper. We address each of their comments in turn below.

**1) Methodology section: Describe how many authors performed the data extraction**.We have now added the following sentence to the methodology section: *Data were extracted by N.J, J.J and R.L.C*. See methods (data extraction and analysis), page 5, paragraph 2

**2) Results section: second paragraph, second sentence: You state key terms were not usually mentioned in relation to a clinical population. We think it would be pertinent to complement this statement by giving a general idea of in what context key terms were generally mentioned in the documents**.Thank you. We have amended the paragraph for clarification: *In most strategies, key terms were not mentioned in the context of specific clinical populations, instead palliative and end of life care was mentioned more broadly for all populations who might require it*. See results, page 6, paragraph 1

**3) Results section: You don’t mention if the strategies make any mention in regards to compassionate communities. It is notable to us as foreign experts that these documents don’t mention them under the governance item, given that the UK is a public health world leader and this is a milestone integration initiative. Maybe it will be worth making a comment in the discussion about this finding**.Thank you for this suggestion. We have added the following into the discussion: *Several strategies mentioned the need to partner with local communities to inform them of and co-produce local palliative and end of life care services. However, there were no mention of ‘compassionate communities’ which recognise that ‘care is not a task solely for health and social care services but is everyone’s responsibility’* (Compassionate Communities 2021 (nihr.ac.uk)*. As outlined in the Lancet commission on the Value of Death, health and social care services should work in partnership with their wider communities to provide care for people at the end of their lives (*Report of the Lancet Commission on the Value of Death: bringing death back into life - The Lancet). *A model of compassionate communities could be utilised by ICSs*. See discussion, page 10, paragraph 2

**4) Results section: The definition of the impact component should be given in the methodological** Thank you for this suggestion. We included the definition of each component in the methods section: *To guide the content analysis, we used and adapted the Social Care Institute for Excellence (SCIE) logic model for integrated care ^14^. We selected this model because it was designed to provide a structure for integrated health and care systems to support planning, performance monitoring, and system improvement ^14^. The original logic model comprised 1) enablers, 2) components, 3) outcomes and 4) impacts. Enablers are described as ‘contextual factors that create the pre-conditions for integrated care’ ^14^. Components of integrated care are described as ‘interventions or activities that create integration’ ^14^. Outcomes reflect the effect of healthcare to improve the health status and wellbeing of patients and informal carers ^16^. Impact is defined by the Social Care Institute for Excellence as ‘long-term benefits that [reflect] the convention of the Triple Aim ^18^ − improving health and wellbeing, enhancing quality and providing best value care’ ^19^. We adapted this model to include Donabedian’s structures and processes ^15^ to extract further detail on the physical components necessary to deliver care and how care is provided and received ^16^. Processes are defined by Donabedian ^16^ as descriptions of how care is provided and received and the ways in which structures are used. Structures are defined by Donabedian as the physical components necessary to deliver care^16^*. See methods (data extraction and analysis), page 5, paragraph 2

**5) Result section: Add the publication years of the ICSs. The time frame of publication of the documents validates their comparability and depicts possible tendencies. Knowing the document’s year of publication could provide an overview of the evolution of these documents towards or against palliative care integration. This information will also better explain your statements on the limitations section where you make a reference to the ICSs recent development albeit the document’s temporality isn’t reported. Further the information in this matter could be easily presented in a figure**.Thank you. We have added in the following information: *We identified strategy documents for 23 of the 42 ICSs (on 30th November 2021) published between 2015 and 2021*. See results, page 5, paragraph 1

**6) Discussion section: On the results you mention only three strategies (15%) state a funding component. The lack of a financial component is a weakness of the strategies, given it has been documented how the competition for resources and their shortage are an obstacle for the implementation of palliative care strategies. We recommend you develop/underline the implication of your result in the discussion section.(DOI:** https://doi.org/10.1016/j.healthpol.2013.07.0161 & DOI:https://doi.org/10.1016/j.healthpol.2022.01.0112) Thank you for this suggestion and for providing references to support the statements made. We have added the following paragraph to the discussion: *Few strategies mentioned the need for adequate funding for staff and services in palliative and end of life care. Palliative care has been shown to be a high value intervention found to reduce reliance on acute hospital care 6– 8. The literature documents that funding for palliative and end of life care needs to be a priority otherwise policy goals and recommendations may not be met (May et al, 2014; Xiao et al, 2022)*. See discussion, page 10, paragraph 4

**7) Discussion section or Strength and limitations section: We think readers will want to replicate your methodology to assess their own country’s policy level of integration. To better explain its usefulness, we think it would be ideal if you compare the pros and cons of your methodology to the ATLANTES’ proposal on palliative care integration assessment. (DOI:**https://doi.org/10.1186/s12904-021-00728-z3 & DOI:https://doi.org/10.3390/ijerph1820107534) Thank you for this suggestion. We have added in the following into the implications section of the paper: *This study was designed to help guide the ongoing development of ICS strategies to strengthen provision of palliative and end of life care as part of integrated care. Our study is timely given the Health and Care Act (2022) now requires ICSs to commission palliative care services (including specialist palliative care) 34. Our adapted logic model can be used to guide the structure of ICS strategies to strengthen commissioning of care for people approaching the end of life. Tools to guide the assessment of palliative care integration into health systems are also available and should be considered by local policy makers (Arias-Casais et al, 2021; Sanchez-Cardenas et al, 2021)*. See implications, page 11, paragraph 1

**8) Conclusion section: We suggest you incorporate the utmost important changes of our feedback in your conclusions**. Thank you. We have amended the conclusion: *Strengthening provision of palliative and end of life care for everyone who needs it is an evidencebased way to both improve care for people approaching the end of life and their families and friends, and to shift the balance of care into the community and reduce pressure on acute services. To support this, ICSs should prioritise and adequately resource palliative and end of life care services. A previous documentary analysis of Health and Wellbeing Strategies in England found that only half mentioned end of life care. The current paper shows some progress, with most ICS strategy documents including aspects of palliative and end of life care that could be mapped onto our adapted logic model. But, for most there was incomplete and poor connection between the different elements. Involving patients and the public in the ongoing development of strategies, using our adapted logic model to ensure focus on the impacts and outcomes that are most important, and identifying evidence-informed interventions that achieve these, will help ensure high quality integrated care for people approaching the end of life. ICSs should continue to monitor palliative care integration into health systems using established tools*. See conclusions, page 11, paragraph 1

Reviewer Report 08 September 2022

https://doi.org/10.21956/amrcopenres.14158.r26991

**© 2022 Scobie S**. This is an open access peer review report distributed under the terms of the Creative Commons Attribution License, which permits unrestricted use, distribution, and reproduction in any medium, provided the original work is properly cited.

✔ **Sarah Scobie**http://orcid.org/0000-0003-0956-5158

Nuffield, Trust, London, UK

Thank you for the opportunity to review this paper, which is timely given the recent launch of integrated care boards, and their statutory duty to plan for end of life care.

The methodology used, analysis and conclusions are reasonable.

I have a few suggestions for amendments which may improve the paper.

Introduction, third paragraph: now that integrated care boards have superseded integrated care systems, the first part of the paragraph needs to move into the past tense (“ICSs were non-statutory…”, “They aimed to…”) and the second part needs to refer to ICBs which are the statutory organisations.

Introduction, fourth paragraph: I would include the timing of the study (“..included in ICS strategy documents, as of the end of November 2021…”. Although it’s in the methods I think this is an important part of the context.

Figure 2: is it possible to improve the resolution and/or text size of this? It is hard to read.

Discussion, second paragraph: I would add here that very few of the plans refer to components which we know are priorities for patients and families, timely access to care (pain relief for example) and 24/7 services. And perhaps point out the lack of focus on PPI shows in the components included in plans. This would then also tie into your paragraph on outcomes.

Strengths and limitations: given the previous analyses of strategies you have done (refs 4 and 12), could you comment here on the extent to which you think the evolving state of ICSs affects the results?

Conclusions: again linking to the previous analyses you have done, could you comment here on whether there has been progress − is there any sign that ICBs might do this better (this might fit better in the implications).

**Is the work clearly and accurately presented and does it cite the current literature?**

Yes

**Is the study design appropriate and is the work technically sound?**

Yes

**Are sufficient details of methods and analysis provided to allow replication by others?**

Yes

**If applicable, is the statistical analysis and its interpretation appropriate?**

Not applicable

**Are all the source data underlying the results available to ensure full reproducibility?**

Yes

**Are the conclusions drawn adequately supported by the results?**

Yes

***Reviewer Expertise:*** Health services research

**I confirm that I have read this submission and believe that I have an appropriate level of expertise to confirm that it is of an acceptable scientific standard**.

Author Response 21 Dec 2022

**Rachel Chambers**, King’s College, London, UK

Thank you to the reviewer for their comments and feedback on our paper. We address each of Sarah’s comments in turn below.

**1) Introduction, third paragraph: now that integrated care boards have superseded integrated care systems, the first part of the paragraph needs to move into the past tense (“ICSs were non-statutory…”, “They aimed to…”) and the second part needs to refer to ICBs which are the statutory organisations**.Thank you for your comment. We have now amended the paragraph which now reads as: *In England, the NHS Long Term Plan (2019) aimed to improve the integration of care through the establishment of Integrated Care Systems (ICSs) ^10^. ICSs were non-statutory partnerships between NHS commissioners, local authorities and other stakeholders who planned health and provided services within specified geographical areas ^11^. They aimed to improve the health of their populations by offering coordinated and efficient services ^11^. In total, 42 ICSs were established, which cover the whole geographical area of England (each with a population size of approximately 1–3 million people) ^11^. ICSs became statutory organisations in July 2022. While there are good reasons for ICSs to include palliative and end of life care in their strategies, previous research has found that palliative care is infrequently a policy priority ^4, 12^*. See introduction, page 2, paragraph 3

**2) Introduction, fourth paragraph: I would include the timing of the study (“..included in ICS strategy documents, as of the end of November 2021…”. Although it’s in the methods I think this is an important part of the context**.We have now added your suggestion to the fourth paragraph: *We aimed to understand whether and how palliative and end of life care was included in ICS strategy documents, and to consider priorities for strengthening this. Searches for ICS strategy documents were completed on 30th November 2021*.See introduction, page 3, paragraph 2

**3) Figure 2: is it possible to improve the resolution and/or text size of this? It is hard to read** We will work with Health Open Research to ensure the resolution and/or text size are improved in the published version.

**4) Discussion, second paragraph: I would add here that very few of the plans refer to components which we know are priorities for patients and families, timely access to care (pain relief for example) and 24/7 services. And perhaps point out the lack of focus on PPI shows in the components included in plans. This would then also tie into your paragraph on outcomes**.Thank you for this suggestion. We have amended the paragraph which now reads as: *Components of care were the most commonly included elements of the adapted logic model, in 18 of 19 strategies. Components focused on allowing patients to have choice and flexibility over their care, through advance care planning, personalised care, and care coordination. This is consistent with previous research which identified effective models of palliative care include: (i) coordination of palliative care across health and social care services; and (ii) involve patients and their families in decision making and goal setting 20. Other components that have been identified as priorities for patients and their families, such as timely access to care and 24/7 services, were not mentioned (Pask et al, 2022). This may reflect the lack of inclusion of patients and the public in the development of ICS strategies*. See discussion, page 8-9, paragraph 2

**5) Strengths and limitations: given the previous analyses of strategies you have done (refs 4 and 12), could you comment here on the extent to which you think the evolving state of ICSs affects the results?** We have added additional detail to the strengths and limitations section: *We did not include analysis of stand-alone palliative and end of life care specific strategies that might exist within the ICSs. Our analysis was completed at a time when ICSs were under formation and were developing their strategies, and it is expected that additional strategies will be published over the next few months. It is also likely that now ICSs are required to commission palliative care services, that their updated or new strategies will include references to palliative and end of life care, which may not have been captured in our analyses. The timing of research such as this is challenging; too soon risks fewer strategies to analyse, whereas too late limits the potential impact to promote changes*. See strengths and limitations, page 10, paragraph 1

**6) Conclusions: again linking to the previous analyses you have done, could you comment here on whether there has been progress − is there any sign that ICBs might do this better (this might fit better in the implications)**.Thank you. We have added additional detail to the conclusions: *Strengthening provision of palliative and end of life care for everyone who needs it is an evidence-based way to both improve care for people approaching the end of life and their families and friends, and to shift the balance of care into the community and reduce pressure on acute services. To support this, ICSs should prioritise and adequately resource palliative and end of life care services. A previous documentary analysis of Health and Wellbeing Strategies in England found that only half mentioned end of life care. The current paper shows some progress, with most ICS strategy documents including aspects of palliative and end of life care that could be mapped onto our adapted logic model. But, for most there was incomplete and poor connection between the different elements. Involving patients and the public in the ongoing development of strategies, using our adapted logic model to ensure focus on the impacts and outcomes that are most important, and identifying evidence-informed interventions that achieve these, will help ensure high quality integrated care for people approaching the end of life. ICSs should continue to monitor palliative care integration into health systems using established tools*. See conclusions, page 11, paragraph 1
