# Supplementary material for: Inclusion of palliative and end of life care in health strategies aimed at integrated care: a documentary analysis [version 2; peer review: 2 approved]
Source: AMRC Open Res. Author manuscript; Available in PMC 2023 Mar 27. (PMC7614372; doi:10.12688/amrcopenres.13079.2)
Supplement: Appendix [file EMS164634-supplement-Appendix.pdf]

Appendix

Appendix 1. NHS Trust or Integrated Care System websites searched for ICS strategy documents.

Integrated Care System	Website
**East of England**	
Bedfordshire, Luton and Milton Keynes	https://blmkhealthandcarepartnership.org/
Cambridgeshire and Peterborough	https://www.cpft.nhs.uk/
Hertfordshire and West Essex	https://www.healthierfuture.org.uk/
Mid and South Essex	https://www.midandsouthessex.ics.nhs.uk/
Norfolk and Waveney	https://www.norfolkandwaveneypartnership.org.uk/
Suffolk and North East Essex	https://www.sneeics.org.uk/
**London**	
North Central London	https://www.northlondonpartners.org.uk/
North East London	https://northeastlondon.icb.nhs.uk/
North West London	https://www.nwlondonccgs.nhs.uk/
South East London	https://www.ourhealthiersel.nhs.uk/
South West London	https://www.swlondon.nhs.uk/

Integrated Care System	Website
**Midlands**	
Birmingham and Solihull	https://www.birminghamandsolihullccg.nhs.uk/
Black Country	https://www.healthierfutures.co.uk/
Coventry and Warwickshire	https://www.happyhealthylives.uk/
Derby and Derbyshire	https://joinedupcarederbyshire.co.uk/
Herefordshire and Worcestershire	https://www.hacw.nhs.uk/
Leicester, Leicestershire and Rutland	https://www.bettercareleicester.nhs.uk/
Lincolnshire	https://www.lincolnshirecommunityhealthservices.nhs.uk/
Northamptonshire	https://www.nhft.nhs.uk/
Nottingham and Nottinghamshire	https://healthandcarenotts.co.uk/
Shropshire, Telford and Wrekin	https://stwics.org.uk/index.php
Staffordshire and Stoke-on-Trent	https://staffsstokeics.org.uk/
**North East and Yorkshire**	
Humber and North Yorkshire	https://humberandnorthyorkshire.org.uk/
North East and North Cumbria	https://www.northcumbriahealthandcare.nhs.uk/
South Yorkshire	https://syics.co.uk/
West Yorkshire	https://www.wypartnership.co.uk/
Cheshire and Merseyside	https://www.cheshireccg.nhs.uk/
Greater Manchester	https://www.gmhsc.org.uk/
Lancashire and South Cumbria	https://www.healthierlsc.co.uk/
**South East**	
Buckinghamshire, Oxfordshire and Berkshire West	https://www.bucksoxonberksw.icb.nhs.uk/
Frimley	www.frimleyhealthandcare.org.uk
Hampshire and the Isle of Wight	https://hiowhealthandcare.org/
Kent and Medway	https://www.kentandmedwayccg.nhs.uk/
Surrey Heartlands	https://www.surreyheartlands.uk/
Sussex	https://www.sussexhealthandcare.uk/
**South West**	
Bath and North East Somerset, Swindon and Wiltshire	https://bswtogether.org.uk/
Bristol, North Somerset and South Gloucestershire	https://bnssghealthiertogether.org.uk/
Cornwall and the Isles of Scilly	https://www.cornwallft.nhs.uk/
Devon	https://www.icsdevon.co.uk/
Dorset	https://ourdorset.org.uk/
Gloucestershire	https://www.onegloucestershire.net/
Somerset	https://nhssomerset.nhs.uk/
